# A Nitric Oxide-Dependent Presynaptic LTP at Glutamatergic Synapses of the PVN Magnocellular Neurosecretory Cells *in vitro* in Rats

**DOI:** 10.3389/fncel.2019.00283

**Published:** 2019-06-27

**Authors:** Bin-Bin Zhang, Hua Jin, Yan-Hua Bing, Xin-Yuan Zhang, Chun-Ping Chu, Yu-Zi Li, De-Lai Qiu

**Affiliations:** ^1^Key Laboratory of Cellular Function and Pharmacology of Jilin Province, Yanbian University, Yanji, China; ^2^Department of Physiology and Pathophysiology, College of Medicine, Yanbian University, Yanji, China; ^3^Department of Nephrology, Affiliated Hospital of Yanbian University, Yanji, China; ^4^Department of Cardiology, Affiliated Hospital of Yanbian University, Yanji, China

**Keywords:** hypothalamic paraventricular nucleus, long-term synaptic plasticity, NMDA receptor, nitric oxide, whole-cell recording, protein kinase A

## Abstract

The magnocellular neurosecretory cells (MNCs) of the hypothalamic paraventricular nucleus (PVN) integrate incoming signals to secrete oxytocin (OT), and vasopressin (VP) from their nerve terminals in the posterior pituitary gland. In the absence of gamma-aminobutyric acid A (GABA_A_) and cannabinoids 1 (CB1) receptor activity, we used whole-cell patch-clamp recording, single-cell reverse transcription-multiplex polymerase chain reaction (SC-RT-mPCR), biocytin histochemistry and pharmacological methods to examine the mechanism of high frequency stimulus (HFS, 100 Hz)-induced long-term potentiation (LTP) at glutamatergic synapses in the PVN MNCs of juvenile male rats. Our results showed that HFS-induced LTP at glutamatergic synapses was accompanied by a decrease in the paired-pulse ratio (PPR) of the PVN MNCs. In these MNCs, HFS-induced LTP persisted in the presence of a group 1 metabotropic glutamate receptor (mGluR1) antagonist; however, it was abolished by an *N*-methyl-D-aspartic acid (NMDA) receptor blocker. Notably, HFS-induced LTP in the PVN MNCs was completely prevented by a nitric oxide synthase (NOS) inhibitor. The application of an NO donor not only induced the LTP of excitatory glutamatergic inputs in the PVN MNCs, but also occluded the HFS-induced LTP in these MNCs. Moreover, HFS-induced LTP in the PVN MNCs was also abolished by a specific protein kinase A (PKA) inhibitor, KT5720. SC-RT-mPCR analysis revealed that 64.5% (62/96) of MNCs expressed OT mRNA. Our results indicate that a HFS can induce an NMDA receptor and NO cascades dependent on presynaptic glutamatergic LTP in the PVN MNCs via a PKA signaling pathway.

## Introduction

The hypothalamic paraventricular nucleus (PVN) is a functionally heterogeneous nucleus consisting of various groups of neurons, which participate in parvocellular neuroendocrine functions (e.g., the stress response and thyroid hormone control) as well as magnocellular neuroendocrine and parvocellular autonomic functions. The three main groups of PVN neurons include the magnocellular neurosecretory cells (MNCs), parvocellular neuroendocrine neurons, and parvocellular pre-autonomic neurons, which synthesize vasopressin (VP), secrete oxytocin (OT) and diverse hypophysiotropic hormones, and control sympathetic nerve activity, respectively (Negoro and Akaishi, 1982; Swanson and Sawchenko, 1983; Kannan et al., 1989; Qiu et al., 2004; Chu et al., 2010, 2013). The MNCs of the hypothalamic PVN integrate incoming information to secrete OT and VP from their nerve terminals in the posterior pituitary (Iremonger et al., 2010). VP is released from the posterior pituitary and controls blood volume by modulating vascular constriction and renal fluid reabsorption. OT is also released into the peripheral blood stream and is essential for milk ejection and parturition (Windle et al., 2004; Rossoni et al., 2008).

Paraventricular nucleus MNCs receive afferent inputs from the median preoptic nucleus (MnPo), subfornical organ (SFO), organum vasculosum of the lamina terminalis (OVLT), brainstem nuclei (Swanson and Sawchenko, 1980), and other intrahypothalamic nuclei (Csaki et al., 2000). These afferent input fibers contain various neurotransmitters, such as excitatory glutamate (van den Pol et al., 1990) and inhibitory gamma-aminobutyric acid (GABA) (Decavel and Van den Pol, 1990). Glutamate is responsible for the majority of fast excitatory neurotransmission (van den Pol et al., 1990), whereas GABA mediates inhibitory transmission in PVN MNCs (Decavel and Van den Pol, 1990). In animals with hypertension, excitatory glutamatergic inputs are a dominant source of excitatory drive to the sympathetic outflow through innervation of the brainstem and spinal cord (Zhou et al., 2018). Activation of the glutamatergic inputs to PVN MNCs induces an increase in spike firing activity via glutamatergic activity enhancement (Li and Ferguson, 1993; Trudel and Bourque, 2010). In addition, the spike firing patterns of MNCs have been reported to be highly dependent on excitatory glutamatergic afferents under *in vitro* and *in vivo* conditions (Iremonger et al., 2010). The application of NMDA can produce repetitive burst-like spike firing activity in the MNCs (Hu and Bourque, 1992; Bains and Ferguson, 1997), whereas spontaneous spike firing activity in OTergic or VPergic neurons is silenced by the application of ionic glutamate receptor antagonists, indicating that this spontaneous spike firing activity is dependent on ongoing glutamatergic action (Nissen et al., 1995; Israel et al., 2003, 2010; Brown, 2004). Presynaptic activity in the OVLT-supraoptic nucleus (SON) pathway increases the rate of spike firing and frequency of excitatory postsynaptic potentials in SON MNCs (Richard and Bourque, 1995). In addition, the brief, high frequency bursts of spike firing exhibited by OTergic neurons prior to milk ejection are thought to result from afferent volleys of glutamatergic excitatory postsynaptic potentials (EPSPs) (Jourdain et al., 1998). Therefore, these excitatory glutamatergic afferents play a critical role in modulating the neurosecretory functions of OTergic and VPergic neurons. In addition, endocannabinoids (eCBs) released from PVN MNCs can transiently depress the release of glutamate from excitatory terminals and influence synaptic plasticity in PVN MNCs (Iremonger et al., 2011).

In the PVN, the actions of glutamate are mediated through ionotropic α-amino-3-hydroxy-5-methyl-4-isoxazolepropionic acid (AMPA), NMDA, and metabotropic glutamate receptors. AMPA and NMDA receptors are activated during fast excitatory synaptic transmission, and generate excitatory postsynaptic currents (EPSCs) at the resting potentials of VP and OT PVN neurons (Wuarin and Dudek, 1993; Stern et al., 1999). NMDA receptors in the PVN play important roles in regulating sympathetic nervous system activation (Martin and Haywood, 1992; Li et al., 2001, 2008), and cardiovascular function (Kawabe et al., 2008). Blocking NMDA receptors inhibits phasic activity in VP neurons and attenuates the spike firing activity of OT neurons during the milk ejection reflex (Nissen et al., 1994; Moos et al., 1997). NMDA receptor-dependent plasticity in the PVN contributed to augmented glutamatergic signaling in spontaneously hypertensive rats (Li et al., 2008). In addition, high frequency stimulation (HFS) of afferent fibers can induce NMDA receptor-dependent long-term potentiation (LTP) of AMPA receptor-mediated EPSCs in rat SON neurons *in vitro*, which suggests that glutamatergic synaptic plasticity in the SON might play an important role in physiological responses to dehydration or lactation (Panatier et al., 2006a,b). Moreover, glutamatergic afferents to PVN parvocellular neurons convey critical excitatory inputs during stress responses and play a critical role in the stress-induced plasticity of corticotropin-releasing hormone-secreting neurons (Salter et al., 2018).

In the brain, neuronal nitric oxide (NO) is an important diffusible second messenger with roles in blood flow regulation, learning and memory, neurotransmitter release, and gene expression (Snyder, 1992; Bruhwyler et al., 1993). Notably, neuronal NO production is thought to depend on NMDA receptor activation (Garthwaite, 2008). In addition to modulating cyclic guanosinc monophosphate (cGMP) pathway, NO can also modulate the cyclic adenosine monophosphate (cAMP)/protein kinase A (PKA) signaling pathway. In rat ventricular myocytes *in vitro*, low NO levels can increase cAMP levels by activating adenylate cyclase (Vila-Petroff et al., 1999). Importantly, NO has been found to mediated long-term synaptic potentiation in other regions of brain, such as cerebellum (Qiu and Knöpfel, 2007; Chu et al., 2014) and hippocampus (Pigott and Garthwaite, 2016). It has been demonstrated that HFS of afferent fibers induced an NMDA receptor-dependent LTP of excitatory glutamatergic inputs in the SON MNCs of rat hypothalamic slices (Panatier et al., 2006a,b), therefore, the NMDA receptors-dependent plasticity in PVN MNCs may be involved in NO production and PKA signaling pathway. However, the NO and PKA signaling cascades in the long-term plasticity in PVN MNCs is currently unknown. In this study therefore we examined the mechanism of HFS-induced LTP at glutamatergic synapses in juvenile rat PVN MNCs in the absence of GABA_A_ and CB1 receptor activity.

## Materials and Methods

### Hypothalamic Slice Preparation

Hypothalamic slices were prepared from P12-14-day-old male Wistar rats, as previously described (Qiu et al., 2004; Chu et al., 2010). The experimental procedures were approved by the Animal Care and Use Committee of Yanbian University and were in accordance with the animal welfare guidelines of the United States National Institutes of Health. The permit number is SYXK (Ji) 2011-006. In brief, the rats were deeply anesthetized with halothane and decapitated quickly. The brain was immediately dissected and placed into ice-cold oxygenated artificial cerebrospinal fluid (ACSF) containing the following (in mM) 125 NaCl, 3 KCl, 1 MgSO_4_, 2 CaCl_2_, 1 NaH_2_PO_4_, 25 NaHCO_3_, and 10 D-glucose bubbled with 95% O_2_/5% CO_2_ (pH 7.3; 295–300 mOsm). Coronal hypothalamic slices (250-μm-thick) were prepared using a vibrating brain slicer (VT 1200s, Leica, Nußloch, Germany). The slices were incubated for at least 1 h in a chamber filled with equilibrated ACSF at room temperature (24–26°C) before electrophysiological recordings were started.

The PVN magnocellular neurons targeted for whole-cell patch-clamp recordings were visualized using a 40× water-immersion lens using a Nikon microscopy (Eclipse FN1, Nikon Corp., Tokyo, Japan). Patch electrodes contained a solution of the following (in mM): potassium gluconate 120, HEPES 10, EGTA 1, KCl 5, MgCl_2_ 3.5, NaCl 4, biocytin 8, Na_2_ATP 4, and Na_2_GTP 0.2 (pH 7.3 with KOH, osmolarity adjusted to 300 mOsm). Patch pipette resistances were 4–6 MΩ in the bath, with series resistances in the range of 10–20 MΩ. Membrane potentials and/or currents were monitored with an Axopatch 700B amplifier (Molecular Devices, Foster City, CA, United States), filtered at 5 kHz, and acquired through a Digidata 1440 series analog-to-digital interface on a personal computer using Clampex 10.4 software (Molecular devices, Foster City, CA, United States). Cells were held in voltage-clamp mode at -70 mV. Series resistance was monitored by applying voltage pulses (10 ms, 5 mV), and only cells with stable series resistance were include in the analysis. For electrical stimulation of excitatory glutamatergic inputs, a stimulating electrode containing ACSF (0.1–0.5 MΩ) was placed in the PVN around magnocellular area of the slice, and paired-current pulses (0.2 ms, 10–100 μA; duration: 50 ms) at 0.05 Hz were delivered through a glass electrode mounted on remote-controlled manipulators (MP-385, Sutter Instrument Company, Novato, CA, United States). The paired-pulse ratio was calculated as the second EPSC (N2) amplitude over the first EPSC amplitude (N1). With exception of Figure 7, the time epoch used for calculate average value of baseline was 10 min before HFS delivering or SNAP administration, while the time epoch used for calculate post-HFS were 40–50 min after HFS or SNAP administration. The amplitude of N1 was normalized by the mean value of baseline.

### Biocytin Histochemistry

After electrophysiology recording, the slice was removed and fixed in 4% paraformaldehyde in 0.1 phosphate buffer (PH 7.4). The slices were incubated over night with the avidin–biotin complex (ABC Elite kit; Vector Laboratories, Burlingame, CA, United States) at room temperature. Finally, biocytin was detected using 3,3′-diaminobenzidine tetrahydrochloride histochemistry.

### Cytoplasm Harvest and Single-Cell Reverse Transcription-Multiplex Polymerase Chain Reaction

Harvesting of cytoplasm and reverse transcription were carried out as previously described (Chu et al., 2013). PCR amplification was performed with a thermal cycler (Mastercycler, nexus gradient; Eppendorf AG, Hamburg, Germany) using a fraction (4 μl) of the single-cell cDNA as a template. First multiplex-PCR was performed as a hot start in final volume of 30 μl containing 4 μl cDNA, 100 pmol of each primer, 0.3 mM of each dNTP, 3 μl 10× PCR buffer, 3.5 U HotStarTaq DNA Polymerase (Takara Bio Inc., Dalian, China) with the following cycling protocol: (1) 15 min at 95°C, (2) 35 cycles of 1 min at 94°C, 1.5 min at 57°C, and 2 min at 72°C, (3) 10 min at 72°C, and then (4) held at 4°C.

The nested-PCR amplifications were carried out in the individual reactions. The following nested primer sequences for GAPDH and OT were used for those neurons: The nested primer sequences were as follows: GAPDH (accession No. NM_017008) external sense: 5′-GATGGTGAAGGTCGGTGTG-3′(position 849), external antisense: 5′-GGGCTAAGCAGTTGGTGGT-3′ (position 1318); GAPDH internal sense: 5′-TACCAGGGCTGCCTTCTCT-3′, internal antisense: 5′-CTCGTGGTTCACACCCATC-3′ (361 bp); OT (accession No. NM_012996) external sense: 5′-ACACACCAGAAGAGGGCATC-3′ (position 1814), external antisense: 5′-GTCAGAGCCAGTAGGCCAAG-3′(position 2580); OT internal sense: 5′-AGGGCCTTTGGTAGAGCAGT-3′, internal antisense: 5′-GAGCTCAAAAGGGACACAGC-3′(416 bp). To investigate the presence and size of the amplified fragments, 10 μl aliquots of PCR products were separated and visualized in an ethidium bromide-stained agarose gel (2%) by electrophoresis. All individual PCR products were verified several times by direct sequencing, using the BigDye Terminator v3.1 Cycle Sequencing Kit and an Applied Biosystems (ABI, Foster City, CA, United States) ABI 3130xl genetic analyzer. Sequence comparison was carried out using the BLAST program. Negative controls which excluded only the harvesting procedure were carried out in parallel to single-cell experiments, and resulted in no detectable bands. The poly (A)+ RNA was prepared from fresh hypothalamus of 13-day-old Wistar rat using Micro-to Midi Total RNA Purification System (Invitrogen). The reverse transcription was performed with 250 μg of the poly(A)+ RNA as described above. The positive controls carried out in parallel with single-cell PCR amplification. The negative controls were carried out in parallel to single-cell experiments excluding only the harvesting procedure, and resulted in no detectable bands (*n* = 10). The SC-RT-mPCR and biocytin staining were performed in the same recorded neuron.

### Chemicals

The reagents, which included N-(piperidin-1-yl)-5-(4-iodophenyl)-1- (2,4-di-chlorophenyl)-4-methyl-1H-pyrazole-3-carboxamide (AM251), cannabinoids type I (CB1) receptors antagonist; (3,4-dihydro-2H-pyrano [2,3-b]quinolin-7-yl)-(*cis-*4-methoxycyclohexyl)-methanone [JNJ16259685 (JNJ)], the group 1 metabotropic glutamate receptor antagonist; *N*^G^-Nitro-L-arginine (L-NNA), NOS inhibitor; *S*-nitroso-*N*-Acetyl-D, L-penicillamine (SNAP), an NO donor. All chemicals were purchased from Sigma-Aldrich (Shanghai, China), while D-APV (D-aminophosphonovaleric acid), a selective NMDA receptor antagonist and KT5720, a specific PKA inhibitor were bought from Tocris Cookson (Bristol, United Kingdom). KT5720 (1 mM) were diluted in dimethyl sulfoxide (DMSO). The experimental concentration of DMSO was less than 0.1% throughout and did not alter or evoke any currents in separate control experiments. In the experiments involving KT5720, the application of KT5720 was started at least 10 min before recording and continuing throughout the experiments. For experiments with L-NNA, the slices were perfused with 200 μM L-NNA for 1 h before recordings were started. The drugs were dissolved in ACSF, and applied directly onto the slices by a peristaltic pump (0.5 ml/min). The ACSF included picrotoxin (50 μM) during all recordings to prevent GABA_A_ receptor-mediated inhibitory, and cannabinoids type 1 (CB1) blocker, AM-251 (1 μM) was included in ACSF during the LTP induction to prevent the CB1 receptor-mediated depression.

### Data Analysis

Electrophysiological data were analyzed using Clampfit 10.3 software (Molecular Devices, Foster City, CA, United States). All data are expressed as the mean ± SEM. One-way ANOVA (*post hoc* multiple comparison) and Mann–Whitney–Wilcoxon test (SPSS software; Chicago, IL, United States) was used to determine the level of statistical significance between groups of data. *P*-values below 0.05 were considered to indicate a statistically significant difference between experimental groups.

## Results

### Blockade of GABA_A_ and CB1 Receptor Activity and HFS-Induced LTP of Excitatory Glutamatergic Inputs in the PVN MNCs

**FIGURE 1:**
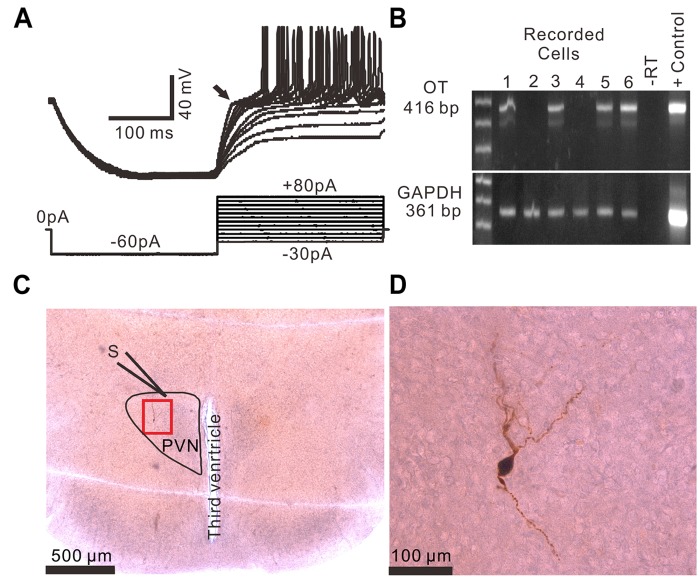
Identification of PVN MNCs. **(A)** Representative membrane potential traces showing a PVN MNC in response to a series of depolarizing injected currents delivered at a hyperpolarized membrane potential, which expressed transient outward rectification (Arrow). **(B)** OT mRNA was detected in the MNCs. The positive control (Tissue Control +) showed that the mRNAs of OT and GAPDH were detected in the rat hypothalamic tissue total RNA. GAPDH transcripts were analyzed in the same cells as an internal control for the RT reaction. The expected size of the PCR products is indicated. A single cell (–RT) and the rat hypothalamic tissue total RNA (Tissue Control +) were processed without RT. **(C)** A histological photo illustrates the morphology of the MNC. S denotes stimulation electrode. **(D)** Enlarged microphotograph of **(C)** showing the morphology properties of the PVN MNCs.

Based on the methods of previous studies (Hoffman et al., 1991; Tasker and Dudek, 1991; Luther et al., 2000; Qiu et al., 2004), 96 PVN neurons from 51 rats were identified as MNCs under whole-cell current-clamp conditions. These putative MNCs expressed a transient outward rectification in response to a series of depolarizing current pulses delivered at a hyperpolarized membrane (Figure 1A). All putative MNCs were screened for GAPDH (positive control) and OT mRNAs using the single-cell reverse transcription-multiplex polymerase chain reaction (SC-RT-mPCR) technique. Rat hypothalamic total RNA (positive control) screening detected GAPDH and OT mRNAs, with each corresponding to the size predicted by its mRNA sequence (Figure 1B). The identities of the PCR fragments were verified by direct sequencing. Of the 96 MNCs, 62 expressed OT mRNA and were classified as OT mRNA-expressing MNCs. Biocytin staining confirmed that these putative MNCs, which had large somas and long dendrites, were PVN MNCs (Figure 1C,D).

**FIGURE 2:**
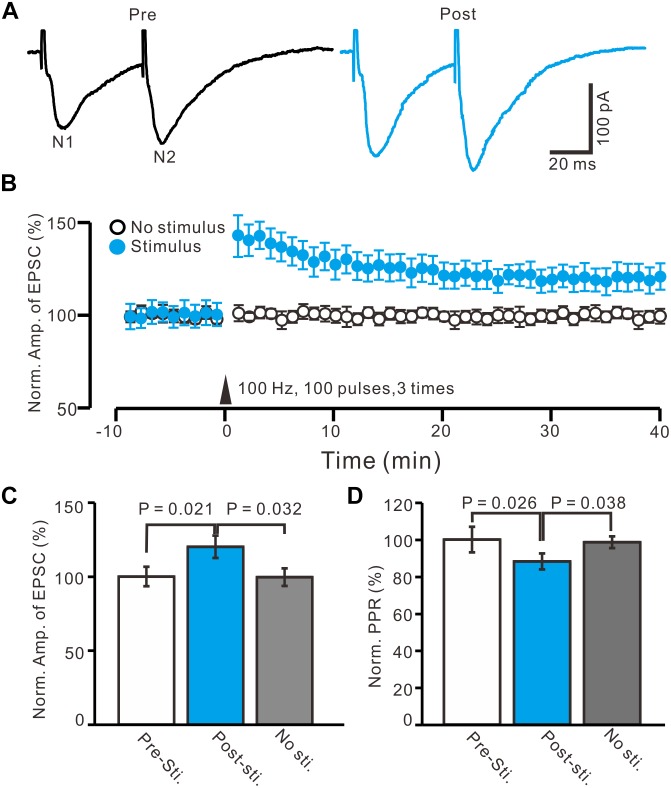
In absence of GABA_A_ and CB1 receptors activity, HFS induced LTP of excitatory glutamatergic inputs in PVN MNCs. **(A)** Representative whole-cell recording traces showing paired-stimulation-evoked EPSCs in a PVN the MNC before (Pre) and after (post) delivering high frequency stimulation (HFS; 100 pulses, 3 times, 10 s interval). **(B)** Summary data showing the time course of the normalized amplitude of N1 under control conditions (No stimulation, open circle; *n* = 10 cells) and delivery of 100 Hz electrical stimulation (arrow head; filled circles; *n* = 16 cells). **(C)** Bar graph (*n* = 16 cells) showing normalized amplitude of N1 before (Pre sti.), after (Post sti.) delivery of HFS and control conditions (No sti.). **(D)** Summary of data (*n* = 16 cells) showing normalized paired-pulse ratio (PPR) before (Pre sti.), after (Post sti.) delivery of 100 Hz stimulation and control conditions (No sti.). Note that electrical stimulation at 100 Hz induced LTP of excitatory glutamatergic inputs accompanied with a decrease in PPR in the PVN MNCs.

In the presence of the GABA_A_ receptor antagonist, picrotoxin (50 μM), and CB1 receptor blocker, AM-251 (1 μM), paired-current pulse (0.2 ms, 10–100 μA; interval, 50 ms) stimulation at 0.05 Hz evoked EPSCs N1 and N2. The average value of N1 was 156.9 ± 6.4 pA, whereas the average value of the paired-pulse ratio (PPR; N2/N1) was 1.35 ± 0.07 (*n* = 96 cells). According to the methods of a previous study (Panatier et al., 2006a,b), we used HFS (100 Hz, 100 pulses, three times) to induce LTP of excitatory inputs in the PVN MNCs (*n* = 16 cells). The HFSs were delivered after acquiring a 10-min control baseline during which the glutamatergic afferent inputs were stimulated at a low frequency (0.05 Hz) to evoke EPSCs (Figure 2A,B). This tetanic stimulation produced persistent potentiation of glutamatergic synaptic transmission, which produced an over 40-min increase in the N1 amplitude (Figure 2B). Between 40–50 min after HFS presentation, the normalized N1 amplitude increased to 120.1 ± 7.5% of the baseline (100.0 ± 6.6%; *P* = 0.021, *n* = 16 cells from 11 mice; Figure 2C). In contrast, under control conditions for 40–50 min, the normalized N1 amplitude was 99.6 ± 5.9% of the baseline (100.0 ± 6.6%; *P* = 0.85, *n* = 10 cells from five mice; Figure 2C), which was significantly lower than that in the HFS group (*P* = 0.032, *n* = 10 cells; Figure 2C). In addition, between 40–50 min after HFS presentation, the normalized PPR decreased to 88.2 ± 4.3% of the baseline (100.0 ± 6.9%; *P* = 0.026, *n* = 16 cells; Figure 2D), whereas, under control conditions for 40–50 min, the normalized PPR was 98.5 ± 3.2% of the baseline (99.9 ± 4.3%; *P* = 0.91, *n* = 10 cells, Figure 2D), which was significantly higher than that in the HFS group (*P* = 0.038, *n* = 16 cells; Figure 2D). These results indicate that electrical stimulation at 100 Hz induced LTP of excitatory glutamatergic inputs as well as a decreased PPR in the PVN MNCs, suggesting that tetanic stimulation induced presynaptic LTP in these MNCs.

### Blocking mGluR1 Receptor Activity Had a Weak Effect on LTP Induction in the PVN MNCs

**FIGURE 3:**
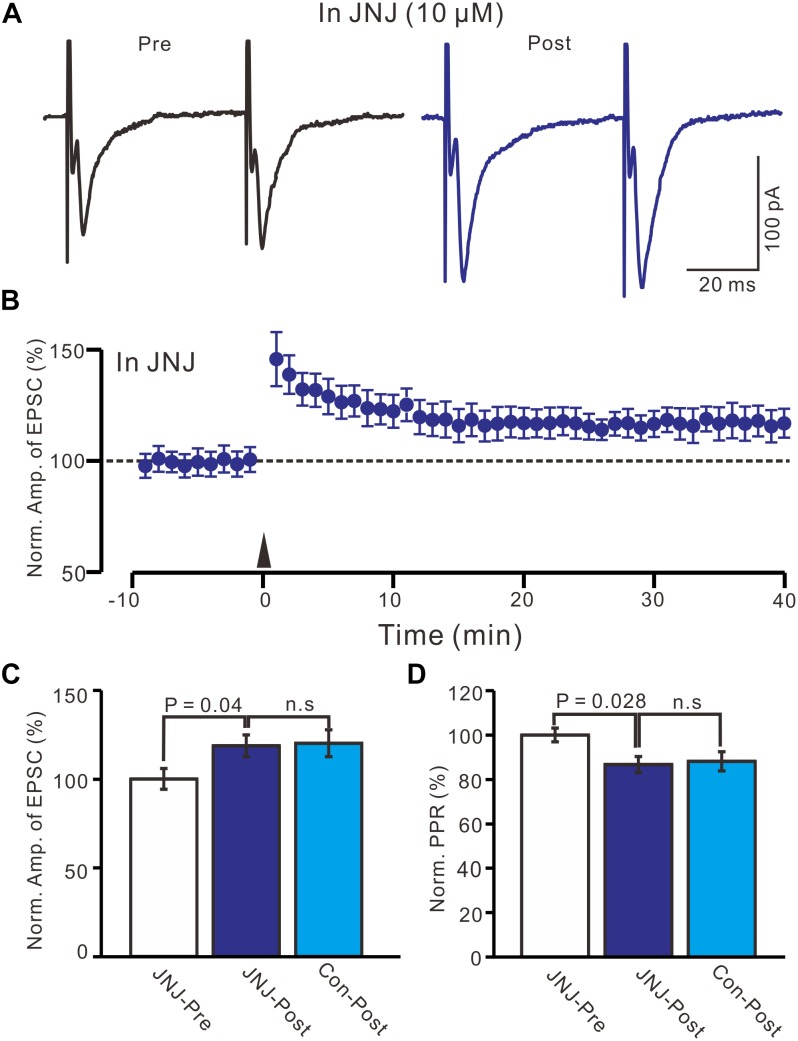
Blockade mGluR1 failed to prevent the LTP induction in the MNCs. **(A)** In the presence of JNJ16259685 (10 μM), representative traces showing paired-pulse stimulation-evoked EPSCs in a PVN MNC before (Pre) and after (post) delivering HFS. **(B)** Summary of data show the time course of the normalized amplitude of N1 before and after delivery of HFS (arrow head; *n* = 12 cells). **(C)** Bar graph (*n* = 12 cells) showing the normalized amplitude of N1 before and after delivery of HFS in the presence of JNJ (JNJ-Pre, JNJ-Post) and control condition (Con-post). **(D)** Summary of data (*n* = 12 cells) showing the normalized PPR before and after delivery of HFS in the presence of JNJ (JNJ-Pre, JNJ-Post) and control condition (Con-post). Note that blockade mGluR1 failed to prevent the LTP induction in the PVN MNCs.

Previous studies have demonstrated that mGluR1 activation is necessary and sufficient for driving retrograde transmitter release and is involved in the synaptic plasticity of PVN neuronal circuitry (Maejima et al., 2001; Chevaleyre and Castillo, 2003; Iremonger et al., 2011). We used an mGluR1 antagonist, JNJ16259685 (10 μM), to examine whether LTP in the PVN MNCs can be induced by mGluR1 activation. In the presence of JNJ16259685, a persistent LTP was still induced in the PVN MNCs (Figure 3A,B). Between 40–50 min after tetanic stimulation and in the presence of JNJ16259685, the normalized N1 amplitude increased to 118.7 ± 6.1% of the baseline (100.0 ± 5.8%; *P* = 0.04, *n* = 12 cells from seven rats; Figure 3C), which was similar to that of the HFS group in ACSF (120.1 ± 7.5% of the baseline; *P* = 0.82, *n* = 16 cells; Figure 3C). Moreover, in the presence of JNJ16259685, the normalized PPR decreased to 86.7 ± 3.6% of the baseline (100.0 ± 3.1%) between 40–50 min after the HFS (*P* = 0.026, *n* = 12 cells; Figure 3D), which was also similar to that of the HFS group in ACSF (88.2 ± 4.3% of the baseline; *P* = 0.68, *n* = 10 cells; Figure 3D). These results indicate that the blockade of mGluR1 activity failed to prevent LTP induction in the PVN MNCs, suggesting that tetanic stimulation-induced presynaptic LTP in these MNCs did not occur because of mGluR1 activation.

### Blocking NMDA Receptor Activity Abolished LTP Induction in the PVN MNCs

**FIGURE 4:**
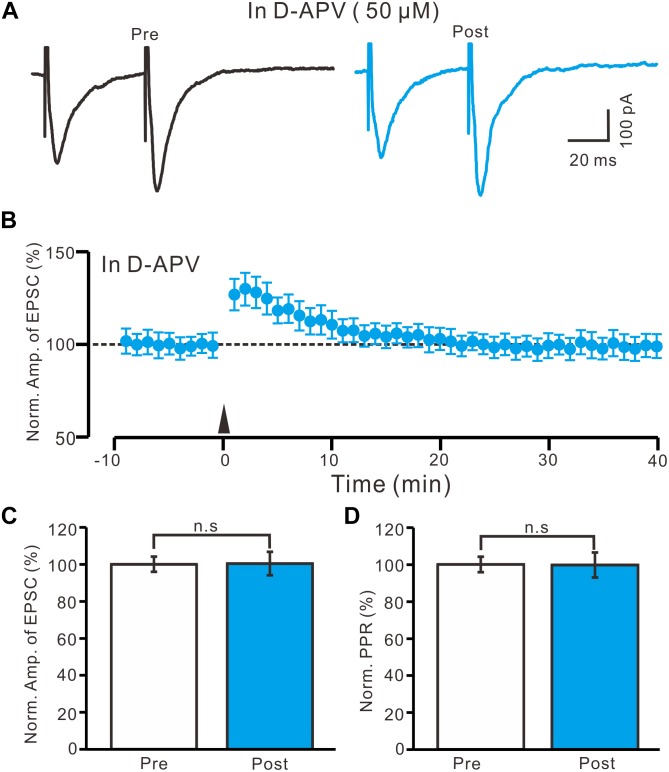
Application of NMDA receptor antagonist abolished the LTP induction in the PVN MNCs. **(A)** In the presence of NMDA receptor blocker, D-APV (50 μM), representative traces showing the stimulation-evoked EPSCs in a PVN the MNC before (Pre) and after (post) delivering HFS. **(B)** Summary of data show the time course of the normalized amplitude of N1 before and after delivery of the HFS (arrow head; *n* = 12 cells). **(C)** Bar graph (*n* = 12 cells) showing the normalized amplitude of N1 before (Pre) and after (Post) delivery of the HFS. **(D)** Summary of data (*n* = 12 cells) showing the normalized PPR before (Pre) and after (Post) delivery of the HFS. Note that NMDA receptor blocker abolished the induction of LTP in the PVN MNCs. Data points are mean ± S.E.M.

High frequency stimulation of afferent fibers caused NMDA receptor-dependent LTP in SON MNCs (Panatier et al., 2006a,b). Thus, we used D-APV (50 μM) to test whether LTP induction in the PVN MNCs occurred via NMDA receptor activation. In the presence of D-APV, the HFS did not induce LTP in the PVN MNCs (Figure 4A,B). Between 40 and 50 min after the HFS, the normalized N1 amplitude was 100.4 ± 6.3% of the baseline (100.0 ± 4.1%; *P* = 0.86, *n* = 12 cells from six rats; Figure 4C). Between 40 and 50 min after tetanic stimulation, the normalized PPR was 99.7 ± 6.8% of the baseline (100.0 ± 4.2%; *P* = 0.81, *n* = 12 cells; Figure 4D). Consistent previous study (Panatier et al., 2006a,b), these results indicate that the blockade of NMDA receptor activity completely prevented LTP induction in the PVN MNCs, suggesting that tetanic stimulation-induced presynaptic LTP in these MNCs was dependent on NMDA receptors.

### HFS-Induced LTP in the PVN MNCs Involves NO

**FIGURE 5:**
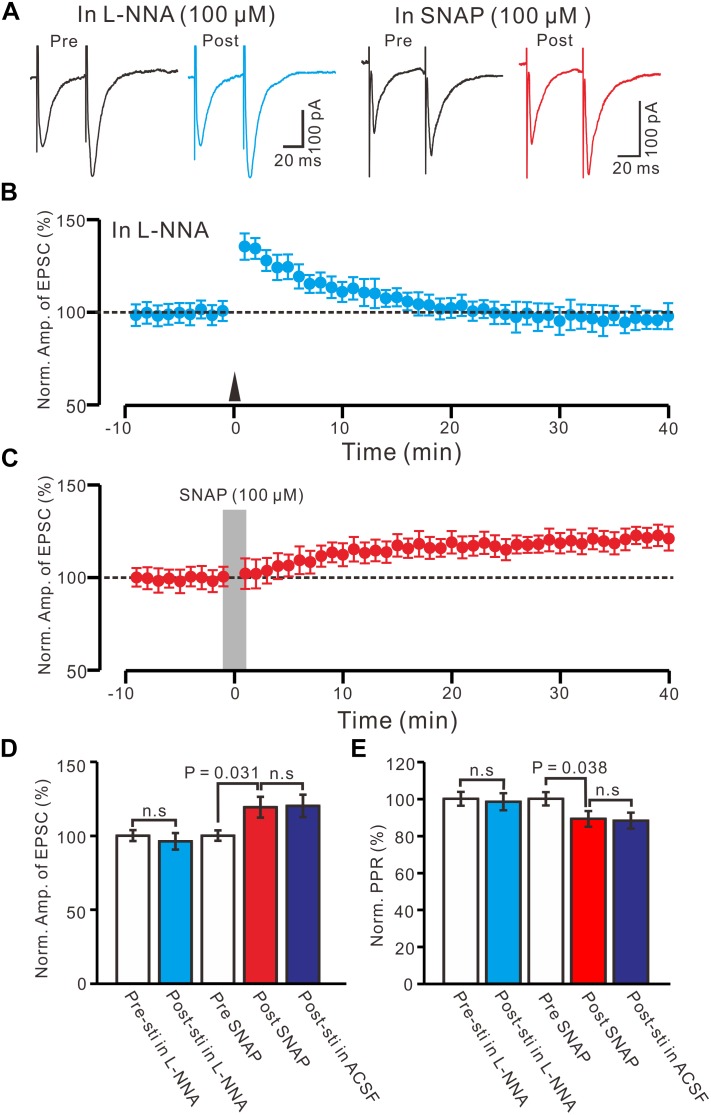
Effect of NOS on the LTP induction in the PVN MNCs. **(A)** Representative traces showing the stimulation-evoked EPSCs in the presence of NOS inhibitor, L-NNA (100 μM), before (Pre) and after (post) delivering the HFS, and before (Pre) and after (post) application of SNAP (100 μM). **(B)** Summary of data show the time course of the normalized amplitude of N1 before and after delivery of the HFS (arrow head; *n* = 12 cells). **(C)** Summary of data show the time course of the normalized amplitude of N1 before (Pre) and after (Post) application of SNAP (100 μM, gray line; 120 s; *n* = 12 cells). **(D)** Bar graph (*n* = 12 cells) showing the normalized amplitude of N1 before (Pre-sti in L-NNA), after delivery of the HFS in L-NNA (Post-sti. in L-NNA), before (Pre SNAP), after application of SNAP (Post SNAP) and after delivery of the HFS in ACSF (Post sti in ACSF). **(E)** Summary of data (*n* = 12 cells) showing the normalized PPR of N1 in each treatment. n.s denotes no significant different.

Nitric oxide is produced by NMDA receptor-dependent nitric oxide synthase (NOS) activation (Garthwaite, 2008), and involved neuronal plasticity in cerebellum (Qiu and Knöpfel, 2007; Chu et al., 2014) and hippocampus (Pigott and Garthwaite, 2016). Therefore, we used a specific neuronal NOS inhibitor, L-NNA (100 μM), to examine whether NO was involved in the LTP of the PVN MNCs. In the presence of L-NNA, the HFS failed to induce LTP in the PVN MNCs (Figure 5A,B). Between 40 and 50 min after tetanic stimulation, the normalized N1 amplitude was 96.2 ± 5.6% of the baseline (100.0 ± 3.7%; *P* = 0.81, *n* = 12 cells from six rats; Figure 5D) and normalized PPR was 98.4 ± 5.2% of the baseline (100.0 ± 3.6%; *P* = 0.72, *n* = 12 cells; Figure 5E). These results indicate that NOS inhibition completely prevented LTP induction in the PVN MNCs, suggesting that HFS-induced presynaptic LTP in these MNCs was influenced by NOS activation.

Furthermore, we examined the effect of an NO donor on the excitatory glutamatergic inputs of the PVN MNCs. Perfusion with SNAP (100 μM) for 2 min induced LTP in the PVN MNCs (Figure 5A,C). Between 40 and 50 min after SNAP application, the normalized N1 amplitude was 119.2 ± 7.1% of the baseline (100.0 ± 3.5%; *P* = 0.031, *n* = 12 cells from six rats), which was similar to that induced by the HFS in ACSF (Figure 5D). The normalized PPR was 89.1 ± 4.2% of the baseline (100.0 ± 3.7%) between 40 and 50 min after the application of SNAP (*P* = 0.038, *n* = 12 cells), which was similar to that induced by the HFS in ACSF (Figure 5E). These results indicate that NOS activation mimicked tetanic stimulation-induced LTP in the PVN MNCs.

**FIGURE 6:**
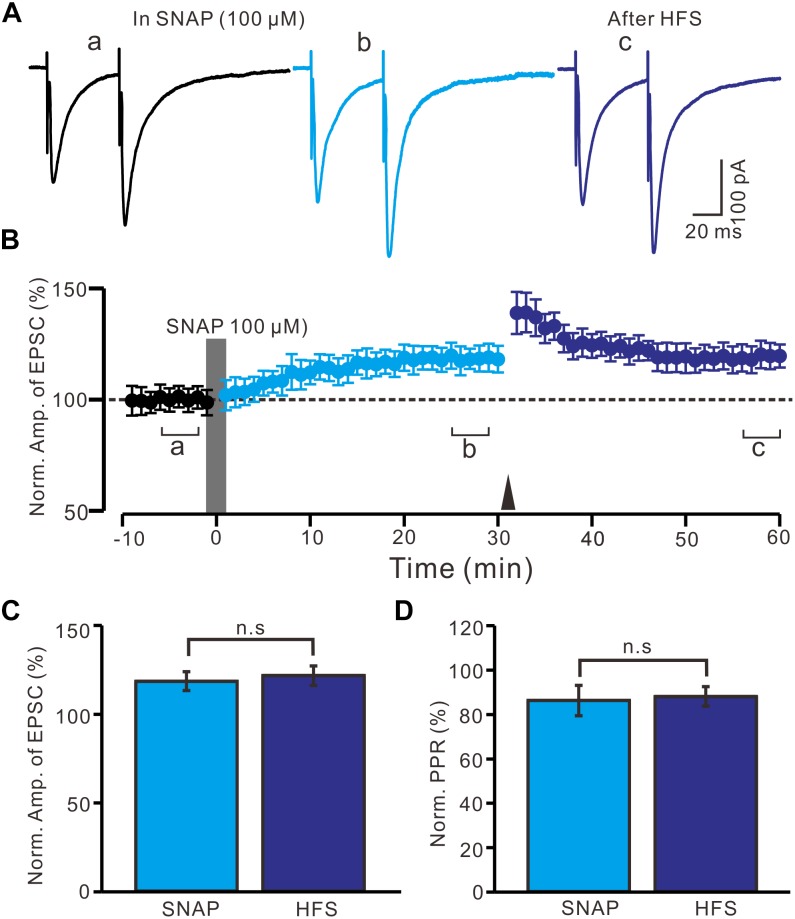
Application SNAP occluded the HFS to induce LTP of excitatory glutamatergic inputs in the PVN MNCs. **(A)** Representative traces showing paired-pulse stimulation-evoked EPSCs in a PVN MNC before (a), after (b) application of SNAP (100 μM), and after delivery of HFS (c). **(B)** Summary of data (*n* = 12 cells) show the time course of the normalized amplitude of N1 before, after application of SNAP (gray line; 120 s) and after delivery of HFS (arrow head). The a, b, and c denote baseline, after application of SNAP and after delivery of HFS, respectively. **(C)** Bar graph (*n* = 12 cells) showing the normalized amplitude of N1 after application of SNAP (SNAP) (b shown in panel **B**) and after delivery of HFS (HFS) (c shown in panel **B**). **(D)** Summary of data (*n* = 6 cells) showing the normalized PPR after application of SNAP (SNAP) and after delivery of HFS (HFS). Note that application NO donor could occlude the HFS to induce LTP of excitatory glutamatergic inputs in the PVN MNCs.

Moreover, we observed whether SNAP-induced LTP could occlude HFS-induced LTP of excitatory glutamatergic inputs in the PVN neurons. SNAP (100 μM) application for 2 min induced LTP in the PVN MNCs. Subsequent HFS delivery produced a transient increase in the N1 amplitude; however, no further LTP induction in the PVN MNCs was observed (Figure 6A,B). The normalized N1 amplitude was 121.3 ± 5.5% of the baseline between 20 and 30 min after HFS delivery, which was similar to that between 20 and 30 min after SNAP application (118.2 ± 5.3% of the baseline; *P* = 0.82, *n* = 12 cells from six rats; Figure 6C). Between 20 and 30 min after HFS delivery, the normalized PPR was 87.9 ± 4.4% of the baseline, which was the same as that between 20 and 30 min after SNAP application (86.1 ± 6.8%; *P* = 0.84, *n* = 12 cells; Figure 6D). These results indicate that NO donor application occluded HFS-induced LTP of excitatory glutamatergic inputs in the PVN MNCs.

### HFS-Induced LTP in the PVN MNCs Occurs Through a PKA Signaling Pathway

**FIGURE 7:**
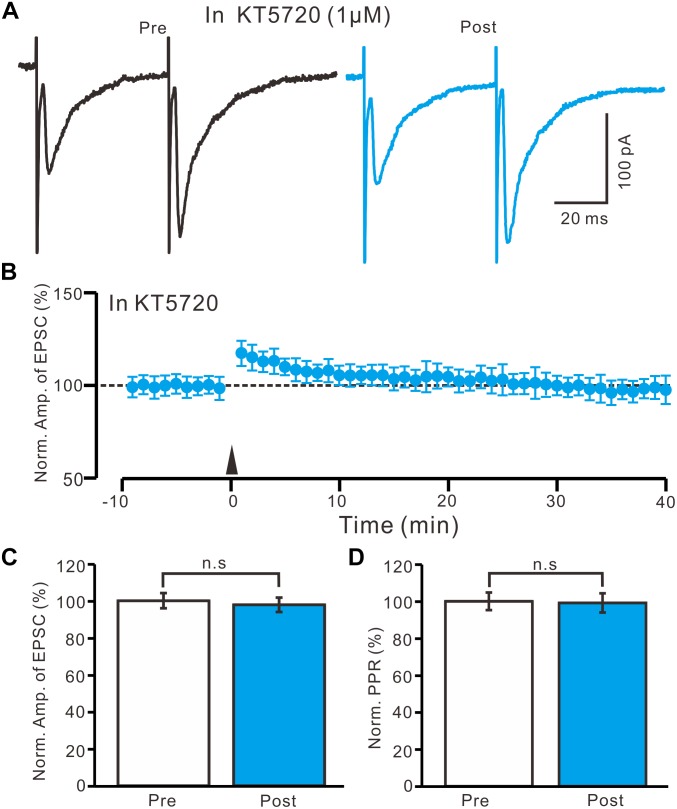
A specific PKA inhibitor, KT5720 completely prevented the LTP induction in the PVN MNCs. **(A)** In the presence of a specific PKA inhibitor, KT5720 (1 μM), representative traces showing paired-pulse stimulation-evoked EPSCs in a PVN MNC before (Pre) and after (Post) delivering the HFS. **(B)** Summary of data show the time course of the normalized amplitude of N1 before and after delivery of the HFS (arrow head; *n* = 10 cells). **(C)** Bar graph (*n* = 10 cells) showing the normalized amplitude of N1 before (Pre) and after (Post) delivery of the HFS. **(D)** Summary of data (*n* = 10 cells) showing the normalized PPR before (Pre) and after (Post) delivery of the HFS. Note that blockade of PKA signaling path way abolished the LTP induction in the PVN MNCs. n.s denotes no significant different.

In the presence of KT5720 (1 μM), HFS delivery failed to induce LTP in the PVN MNCs (Figure 7A,B). Between 40 and 50 min after tetanic stimulation, the normalized N1 amplitude was 97.8 ± 3.9% of the baseline (100.0 ± 4.1%; *P* = 0.78, *n* = 10 cells from five rats; Figure 7C) and normalized PPR was 99.1 ± 5.2% of the baseline (100.0 ± 4.8%; *P* = 0.84, *n* = 10 cells; Figure 7D). These results indicate that the blockade of a PKA signaling pathway abolished LTP induction in the PVN MNCs, suggesting that the HFS induced presynaptic LTP in these MNCs via a PKA signaling pathway.

## Discussion

Our main finding is that, in the absence of GABA_A_ and CB1 receptor activity, HFS can induce an NMDA receptor- and NO- dependent presynaptic glutamatergic LTP in the PVN MNCs through the PKA signaling pathway.

### HFS-Induced LTP at Excitatory Glutamatergic Synapses in the PVN MNCs Without GABA_A_ and CB1 Receptor Activity

Both PVN OTergic and VPergic neurons receive information from excitatory glutamate inputs (van den Pol et al., 1990) and inhibitory GABAergic afferents (Decavel and Van den Pol, 1990; Zhou et al., 2018). Glutamate is responsible for the majority of fast excitatory neurotransmission, which plays a critical role in controlling OTergic and VPergic neurons activity (van den Pol et al., 1990), whereas GABA is responsible for the majority of fast inhibitory neurotransmission, which modulates OTergic neuronal excitability (Decavel and Van den Pol, 1990). Under *in vivo* conditions, pharmacological NMDA receptor activation produces repetitive, burst-like discharges within MNCs, which suggests that activation of glutamatergic input can increase the neuronal activity of MNCs (Hu and Bourque, 1992; Bains and Ferguson, 1997). In our study, electrical stimulation of excitatory glutamate inputs evoked EPSCs with paired-pulse facilitation in the absence of GABAergic inhibition, which confirms that excitatory glutamate inputs synapse onto OTergic and VPergic MNCs of PVN from juvenile rats (Li and Ferguson, 1993; Trudel and Bourque, 2010). Our results suggest that presynaptic LTP of MNCs through glutamatergic inputs might play a crucial role in controlling electrical behavior and, consequently, the secretory activity of PVN OT and VP neurons in juvenile rats.

A previous study demonstrated that HFS of afferent fibers could induce LTP of AMPA receptor-mediated EPSCs in rat SON neurons under *in vitro* conditions (Panatier et al., 2006a,b). Our present results show that electrical stimulation at 100 Hz induced LTP at the excitatory glutamatergic inputs of the PVN MNCs in juvenile rats. Notably, HFS-induced LTP showed a decrease in the PPR, indicating a change in the presynaptic release probability. This result suggests that HFS-induced LTP in the PVN MNCs was presynaptically expressed (Qiu and Knöpfel, 2007; Chu et al., 2014). In the hypothalamic PVN, glutamate afferents synapse onto parvocellular neurons, which convey critical excitatory inputs during stress and undergo stress-induced plasticity in corticotrophin-releasing hormone-secreting neurons (Salter et al., 2018). Moreover, eCBs are released from hypothalamic PVN MNCs following coincident bursts of pre- and postsynaptic spiking, which transiently depress the release of glutamate from excitatory terminals and, in turn, prevent synaptic long-term depression (LTD) (Iremonger et al., 2011). Therefore, we studied HFS-induced LTP at excitatory glutamatergic inputs in the PVN MNCs from juvenile rats in the absence of CB1 receptor activity. However, the magnitude of LTP observed in the present study is smaller than that observed in SON MNCs (Panatier et al., 2006a,b). Differential MNC LTP expression between the PVN and SON might be related to their distinct excitatory glutamatergic inputs.

The SC-RT-PCR results showed that over 60% of the recorded MNCs expressed OT mRNA, suggesting that HFS-induced LTP at excitatory glutamatergic inputs in the PVN OT mRNA-expressing MNCs from juvenile rats. However, the PVN MNCs include OTergic and VPergic MNCs, therefore the other MNCs did not express OT mRNA are reasonably expected to be VPergic neurons. In addition, it has been demonstrated that the majority of MNCs in the SON and PVN co-express both OT and VP mRNA (da Silva et al., 2015; Dos-Santos et al., 2018). Therefore, our results indicate that the HFS induces presynaptic LTP in PVN MNCs, which may include both OTergic and VPergic neurons.

### LTP Induction in the PVN MNCs Depends on an NMDA Receptor/NO/PKA Signaling Pathway

Paraventricular nucleus NMDA receptors play important roles in regulating sympathetic nervous system activation (Martin and Haywood, 1992; Li et al., 2001, 2008), and cardiovascular function (Kawabe et al., 2008). NMDA receptor blockade inhibits phasic activity in MNCs and attenuates the spike firing activity of OT neurons during the milk ejection reflex (Nissen et al., 1994; Moos et al., 1997), whereas NMDA receptor activation produces repetitive burst-like discharges in MNCs (Hu and Bourque, 1992; Bains and Ferguson, 1997). Therefore, NMDA receptors play a critical role in controlling MNC activity in the hypothalamic PVN. Notably, studies of the SON (Panatier et al., 2006a,b) and other brain regions (Malenka and Nicoll, 1999; Malenka and Bear, 2004) have shown that NMDA receptors play an important role in the induction of long-term synaptic changes (e.g., LTP and LTD dependence on NMDA receptors). NMDA receptor-dependent plasticity in the PVN contributes to augmented glutamatergic signaling in spontaneously hypertensive rats (Li et al., 2008). Consistent with previous SON studies (Panatier et al., 2006a,b; Iremonger et al., 2011), we found that the blockade of NMDA receptor activity completely prevented LTP induction in the PVN MNCs. This finding suggests that HFS-induced presynaptic LTP in the PVN MNCs was dependent on NMDA receptors. In addition, our results showed that an mGluR1 activity blockade failed to prevent LTP induction in the PVN MNCs, which suggests that the tetanic stimulation-induced presynaptic LTP in these MNCs was unrelated to mGluR1 activation.

Our results showed that HFS-induced LTP in the PVN MNCs was prevented by an NOS inhibitor and blocked by a specific PKA inhibitor. These results suggest that the HFS induced NO cascade- and PKA signaling-dependent LTP in the PVN MNCs. NO can be produced by NMDA receptor-dependent activation of neuronal NOS (Garthwaite, 2008). The observation of NMDA receptor 1 in a subset of PVN dendrites containing neuronal NOS and receiving excitatory-type synaptic contacts is consistent with the well-characterized spatial coupling of NMDA receptors and neuronal NOS in the hypothalamus (Bhat et al., 1995; Kiss and Vizi, 2001). In the PVN, OT, and VP neurons are the major neuronal phenotypes that express neuronal NOS (Hatakeyama et al., 1996; Gonza´lez-Herna´ndez et al., 2006). Direct NO or NO donor administration into the PVN decreases sympathetic nerve activity and lowers arterial blood pressure (Horn et al., 1994; Zhang and Patel, 1998). Our results indicate that NOS inhibition completely prevented LTP induction in the PVN MNCs, which suggests that HFS-induced presynaptic LTP in these MNCs was involved in NOS activation. Furthermore, we found that SNAP perfusion also induced LTP in the PVN MNCs and confirmed that NOS activation mimicked HFS-induced LTP in these MNCs. NO donor application occluded the HFS-induced LTP of excitatory glutamatergic inputs in the PVN MNCs, which confirmed that SNAP and the HFS were using the same signaling pathway. Under *in vitro* conditions, NMDA receptor activation increased calcium influx and consequent intracellular cGMP accumulation, resulting in NOS activity enhancement in cerebellar granule cells (Kiedrowski et al., 1992). Consistent with that study (Kiedrowski et al., 1992), our results suggest that HFS delivery activated NMDA receptors, which enhanced NO production in the PVN MNCs of juvenile male rats.

In addition, our results showed that the PKA signaling pathway blockade abolished LTP induction in the PVN MNCs, suggesting that tetanic stimulation induced presynaptic LTP in these MNCs via a PKA signaling pathway. In rat ventricular myocytes, low NO levels can increase intracellular cAMP concentrations through adenylate cyclase activation, which indicates that NO can modulate the cAMP/PKA signaling pathway (Vila-Petroff et al., 1999). Moreover, NO is produced in an activity-dependent manner in response to increases in intracellular calcium during repetitive spike firing (Stern and Ludwig, 2001). Therefore, our results suggest that a HFS might increase NO levels via NMDA receptor activation and lead to LTP and glutamate release in OTergic and VPergic neurons through the cAMP-PKA signaling pathway. Taken together, our study demonstrates that a HFS can induce an NMDA receptor and NO cascades dependent on presynaptic glutamatergic LTP in the PVN MNCs through the PKA signaling pathway. These findings suggest that presynaptic glutamatergic LTP in the PVN MNCs could play an important role in physiological responses to stress and anxiety.

## Conclusion

In the present study, we found that HFS induced LTP at glutamatergic synapses accompanied with a decrease in paired-pulse ratio in the PVN MNCs. The HFS-induced LTP in the PVN MNCs was persistent in the presence of group 1 metabotropic glutamate receptor antagonist, but it was not induced in the absence of NMDA receptors activity. The HFS-induced LTP in the PVN MNCs abolished by nitric oxide synthase inhibitor, L-NNA, and could be induced by perfusion of NO donor, SNAP. In the presence of a specific protein kinase A inhibitor, KT5720, delivery of HFS failed to induce LTP in the PVN MNCs. These results indicated that presynaptic glutamatergic LTP in the PVN MNCs could play an important role in the context of physiological responses, such as stress and anxiety.

## Ethics Statement

The experimental procedures were approved by the Animal Care and Use Committee of Yanbian University and were in accordance with the animal welfare guidelines of the United States National Institutes of Health. The permit number is SYXK (Ji) 2011-006.

## Author Contributions

All authors listed have made a substantial, direct and intellectual contribution to the work, and approved it for publication.

## Conflict of Interest Statement

The authors declare that the research was conducted in the absence of any commercial or financial relationships that could be construed as a potential conflict of interest.
